# The Impact of COVID-19 on the mental health of dialysis patients

**DOI:** 10.1007/s40620-021-01005-1

**Published:** 2021-03-19

**Authors:** Anna A. Bonenkamp, Theresia A. Druiventak, Anita van Eck van der Sluijs, Frans J. van Ittersum, Brigit C. van Jaarsveld, Alferso C. Abrahams, the DOMESTICO study group

**Affiliations:** 1 Department of Nephrology, Amsterdam UMC, Vrije Universiteit Amsterdam, Research Institute Amsterdam Cardiovascular Sciences, Amsterdam, Netherlands; 2 Department of Nephrology and Hypertension, University Medical Centre Utrecht, Utrecht, The Netherlands; 3 Diapriva Dialysis Centre, Amsterdam, The Netherlands

**Keywords:** COVID-19, Mental health, Health-related quality of life, Chronic dialysis

## Abstract

**Background:**

Studies have shown increased anxiety, depression, and stress levels among different populations during the coronavirus disease 2019 (COVID-19) pandemic. However, the impact of the pandemic on the mental health of dialysis patients remains unknown. The aim of this study was to investigate the mental health of dialysis patients during the COVID-19 pandemic compared to the period preceding the pandemic.

**Methods:**

Data originate from the ongoing multicentre observational Dutch nOcturnal and hoME dialysis Study To Improve Clinical Outcomes (DOMESTICO). Patients who filled in a health-related quality of life (HRQoL) questionnaire during the pandemic and six to three months prior were included. The mean difference in Mental Component Summary (MCS) score of the Short Form 12 (SF-12) was analysed with multilevel linear regression. A McNemar test was used to compare presence of mental health-related symptoms during and prior to the COVID-19 pandemic.

**Results:**

A total of 177 patients were included. The mean MCS score prior to COVID-19 was 48.08 ± 10.15, and 49.00 ± 10.04 during the COVID-19 pandemic. The adjusted mean MCS score was 0.93 point (95% CI − 0.57 to 2.42) higher during the COVID-19 pandemic than during the period prior to the pandemic. Furthermore, no difference in the presence of the following mental health-related symptoms was found during the COVID-19 pandemic: feeling anxious, feeling sad, worrying, feeling nervous, trouble falling asleep, and trouble staying asleep.

**Conclusions:**

The mental health of dialysis patients appears to be unaffected by the COVID-19 pandemic. Dialysis patients may be better able to cope with the pandemic, since they have high resilience and are less impacted by social distancing measures.

**Trial registration number:**

Netherlands Trial Register NL6519, date of registration: 22 August 2017.

**Graphic abstract:**

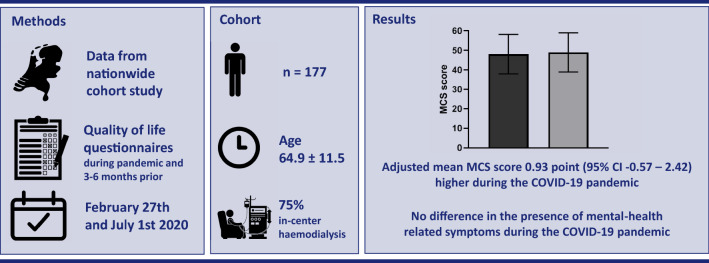

**Supplementary Information:**

The online version contains supplementary material available at 10.1007/s40620-021-01005-1.

## Introduction

The coronavirus disease 2019 (COVID-19) outbreak that started in China rapidly spread across the globe, with major consequences for health and healthcare systems. Currently, the estimated number of infections worldwide is 66 million and the estimated number of deaths is 1.5 million [1]. In the Netherlands, the first COVID-19 patient was diagnosed on February 27th, 2020 [2]. In response, the Dutch government announced drastic measures; they obliged social distancing including working from home and closing all educational institutions, restaurants, cultural and sporting facilities, to limit further spread of the virus.

The current COVID-19 outbreak has been shown to increase levels of anxiety, depression, and stress among the general population [3–5]. In patients with Alzheimer’s disease and immunodeficiency, COVID-19 also resulted in higher anxiety levels and a higher risk of developing depression [6, 7]. Moreover, patients with chronic conditions had an increased risk of developing sleeping disorders [8]. Patients with end-stage kidney disease (ESKD) who are treated with dialysis have a higher risk of a severe clinical course of COVID-19 and worse outcome [9]. The knowledge that they have a higher risk of infection, can become more seriously ill and have a higher mortality risk might result in symptoms like feeling anxious, feeling sad, worrying, feeling nervous and sleeping problems. Moreover, the psychological well-being of dialysis patients may also be affected by fear among fellow patients and healthcare professionals. However, data regarding the impact of the COVID-19 pandemic on the mental health of dialysis patients are lacking. The aim of this study was to investigate the mental health of dialysis patients during the COVID-19 pandemic compared to the period preceding the pandemic.

## Methods

### Study population and design

To compare the mental health of dialysis patients prior to the COVID-19 pandemic with a period during the COVID-19 pandemic, data were used from the ongoing Dutch nOcturnal and hoME dialysis Study To Improve Clinical Outcomes (DOMESTICO, Netherlands Trial Register identifier: NL6519) [10]. In this nationwide, prospective, observational study the health-related quality of life (HRQoL) of home dialysis, i.e. peritoneal dialysis and home haemodialysis, patients is compared with the HRQoL of in-centre haemodialysis patients. All adult patients that started chronic dialysis were potentially eligible and all included patients provided written informed consent. The first patient was recruited in December 2017 and the end of the inclusion period is expected in 2021.

For the present study, patients were included if they had completed a HRQoL questionnaire during the COVID-19 pandemic, defined as the period between February 27th and July 1st, 2020, and a questionnaire 6 months prior to the COVID-19 pandemic. When the questionnaire administered 6 months prior to the COVID-19 pandemic was not available, the questionnaire administered 3 months prior to the COVID-19 pandemic was used.

### Outcome parameters

The primary outcome parameter was mental health, assessed with the Mental Component Summary (MCS) score of the 12-item Short Form (SF-12) health survey. The MCS was calculated using standard algorithms, meaning that a healthy individual scores 50 points on a scale of 0–100 with a standard deviation of 10 points [11, 12]. Higher scores of the MCS reflect better HRQoL [11]. The secondary outcome parameters were the Physical Component Summary (PCS) score of the SF-12, and the presence and severity of mental health-related symptoms assessed with the Dialysis Symptom Index (DSI) [11, 13]. These symptoms included feeling anxious, feeling sad, worrying, feeling nervous, trouble falling asleep, and trouble staying asleep. A 5-point Likert scale, ranging from ‘not at all bothersome’ to ‘very bothersome’, was used to evaluate the severity of these 6 symptoms [13].

### Data collection

The following sociodemographic and clinical data were collected at study baseline: sex, age, primary kidney disease, living situation (alone, with a partner, or in a nursing home), level of education, work status, history of comorbidities, recent start, dialysis modality (in-centre haemodialysis, peritoneal dialysis, or home haemodialysis), and acute start at dialysis initiation. Primary kidney disease was classified according to the codes of the ERA-EDTA. A higher level of education includes university colleges and university of applied sciences. Comorbidity was scored according to the Charlson comorbidity index [14]. Recent start of dialysis was defined as start of dialysis 6 months prior to the COVID-19 pandemic. Acute start of dialysis was defined as an unplanned start of dialysis with no previous consultation of a nephrologist.

In addition, the questionnaires were reviewed to check whether participants had written comments related to COVID-19.

### Statistical analysis

All normally distributed continuous variables are presented as mean with standard deviation (SD), non-normally distributed variables as median with interquartile range (IQR), and categorical variables as proportion.

Multilevel linear regression was used to assess the overall association between the COVID-19 pandemic and MCS or PCS score. The multilevel model was used to adjust for correlation of repeated observations within a patient. Both crude and adjusted analyses were performed. Adjusted models were corrected for sex, age, Charlson comorbidity index, higher education level, dialysis modality, and recent start of dialysis.

A McNemar test was used to compare the presence of mental health-related symptoms prior to the COVID-19 pandemic with the period during the COVID-19 pandemic. A Wilcoxon signed-rank test was used to compare the severity of mental health-related symptoms prior to the COVID-19 pandemic with the period during the COVID-19 pandemic. In addition, the severity scores of the 6 mental health-related symptoms were added up to an overall symptom severity score ranging from 0 to 30, in which a severity score of 30 meant that in all mental health-related symptoms the maximum severity score was reported [13, 15].

Missing values of SF-12 items and confounders were imputed with standard multiple imputation techniques using 10 repetitions and predictive mean matching (SPSS) [16]. A difference of 3 points on the MCS and PCS was considered clinically relevant and a p-value of < 0.05 was considered statistically significant [17, 18]. All analyses were performed using SPSS Statistics version 26 (IBM) or STATA 14.

## Results

A total of 177 patients were included, of whom 125 had filled in a questionnaire 6 months prior to the COVID-19 pandemic and 52 had filled in a questionnaire 3 months prior to the COVID-19 pandemic. The majority of patients (87%) had filled in their HRQoL questionnaires completely. Patient characteristics are depicted in Table 1. The majority (63%) was male, the mean age of the study population was 64.9 ± 11.5 years and 61% started dialysis 3–6 months prior to the COVID-19 pandemic. Only 1% of the study population was infected with SARS-CoV-2.

**Table 1:** Patient characteristics

Characteristics	Patients (*n* = 177)
Sex, male, *n* (%)	112 (63)
Age, mean (SD), years	64.9 ± 11.5
Primary kidney disease, *n* (%)	
Glomerulonephritis/pyelonephritis	27 (21)
Cystic kidney disease	12 (9)
Renovascular kidney disease	28 (21)
Diabetes mellitus	24 (18)
Other/unknown	41 (31)
Living situation, *n* (%)	
Alone	49 (31)
With a partner	95 (60)
In a nursing home	4 (3)
Higher education, *n* (%)	34 (21)
Employed, *n* (%)	27 (16)
Charlson comorbidity index, median [IQR]	4 [2–5]
Recent start of dialysis, *n* (%)	107 (61)
Dialysis modality at dialysis initiation, *n* (%)	
In-centre haemodialysis	132 (75)
Peritoneal dialysis	43 (25)
Home haemodialysis	2 (1)
Acute start of dialysis, *n* (%)	25 (14)
Infected with SARS-CoV-2, *n* (%)	2 (1)

**Fig. 1:**
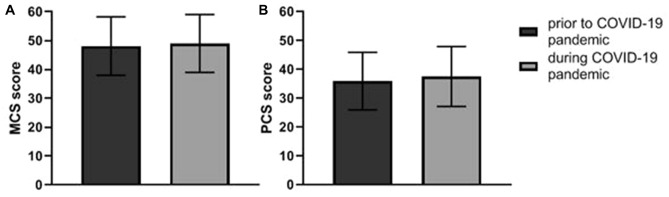
Mental Component Summary score (**a**) and Physical Component Summary score (**b**) prior to and during the COVID-19 pandemic. *MCS* mental component summary, *PCS* physical component summary

The MCS score was 48.08 ± 10.15 prior to the COVID-19 pandemic and 49.00 ± 10.04 during the COVID-19 pandemic (Fig. 1a). The mean MCS score was 0.91 point (95% CI − 0.59 to 2.41, *p*-value 0.2) higher during the COVID-19 pandemic than prior to the COVID-19 pandemic (Table 2). Adjustment for multiple confounders did not change this result.

**Table 2:** Linear regression of Health-Related Quality of Life score during the COVID-19 pandemic

	regression coefficient (95% CI)		
	Crude	Adjusted^a^	Adjusted^b^
MCS change during COVID-19	0.91 (− 0.59 to 2.41)	0.91 (− 0.59 to 2.41)	0.93 (− 0.57 to 2.42)
PCS change during COVID-19	1.63 (0.28 to 2.99)	1.63 (0.28 to 2.98)	1.64 (0.28 to 2.99)

*MCS* mental component summary, *PCS* physical component summary

^a^Adjusted for age and sex

^b^Adjusted for age, sex, Charlson comorbidity index, higher education level, dialysis modality, and recent start of dialysis

The PCS score was 35.92 ± 9.99 prior to the COVID-19 pandemic and 37.52 ± 10.38 during the COVID-19 pandemic (Fig. 1b). The mean PCS score was 1.63 point (95% CI 0.28 to 2.99, *p*-value 0.02) higher during the COVID-19 pandemic than prior to the COVID-19 pandemic (Table 2). Adjustment for multiple confounders did not change this result.

**Fig. 2:**
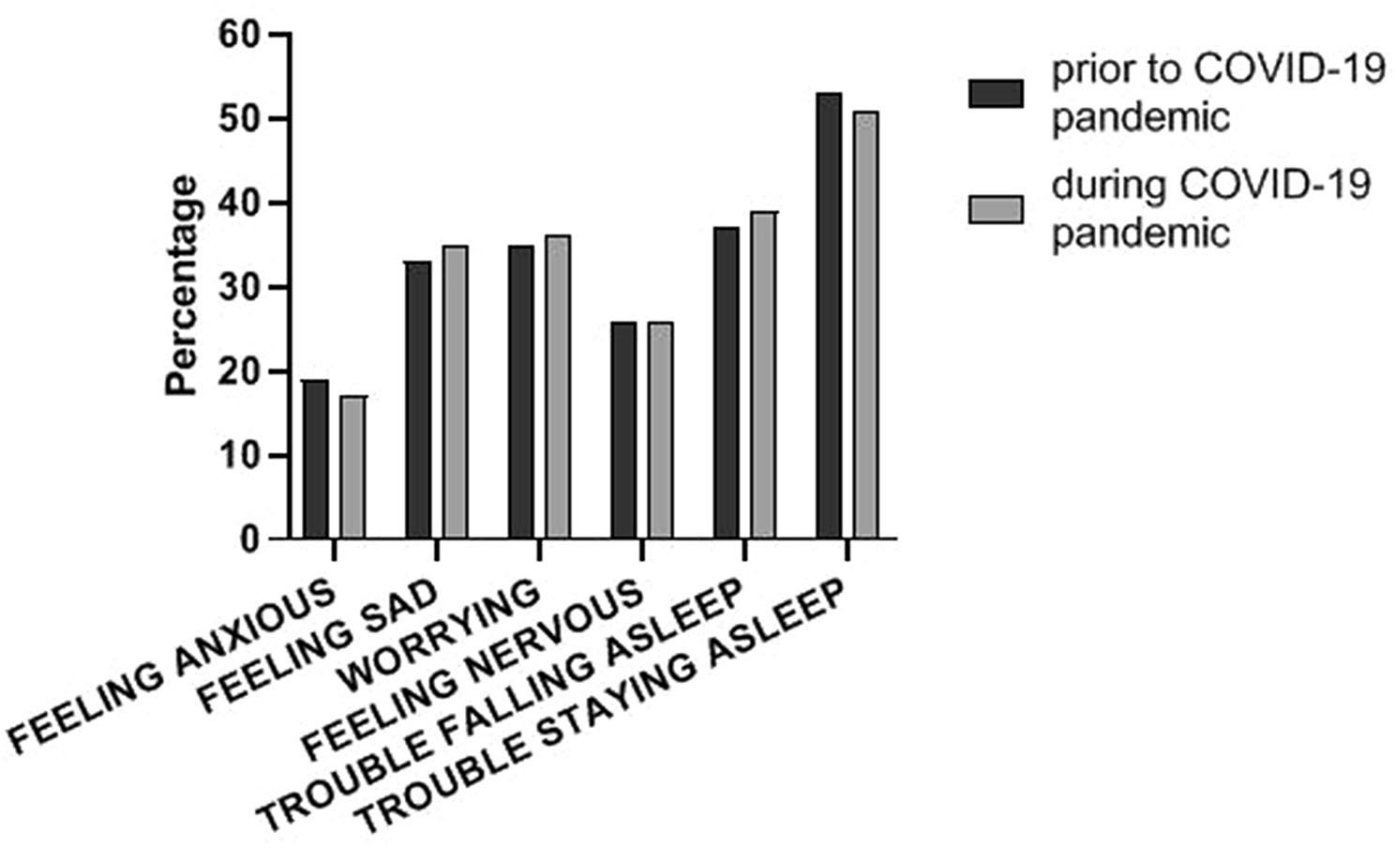
Presence of mental health-related symptoms prior to and during the COVID-19 pandemic

As depicted in Fig. 2, patients on dialysis reported frequently that they were feeling sad (33% vs 35%), were worrying (35% vs 36%), had trouble falling asleep (37% vs 39%) and had trouble staying asleep (53% vs 51%). For all mental health-related symptoms, there was no significant difference in presence prior to the COVID-19 pandemic compared to the period during the COVID-19 pandemic.

Also, no difference was found regarding the total number of mental health-related symptoms: 74% of patients reported at least 1 symptom prior to the COVID-19 pandemic compared to 72% of patients during the COVID-19 pandemic, while 7% of patients reported all 6 symptoms prior to the COVID-19 pandemic compared to 6% of patients during the COVID-19 pandemic.

The severity of mental health-related symptoms was not significantly higher during the COVID-19 pandemic (Supplementary Fig. 1). In addition, the total symptom severity score (ranging from 0 to 30) did not differ between the two time periods (4 [IQR 0–8] prior to the pandemic vs 4 [IQR 0–9] during the COVID-19 pandemic).

Finally, a few patients wrote comments on the questionnaire concerning the COVID-19 pandemic. Patients commented that the COVID-19 pandemic had a huge impact on everyday life and that more help by informal caregivers was needed. For example, a patient wrote ‘this corona period also affects our daily life. Due to my health condition, we have tried to avoid all threats. The domestic help is no longer coming and grocery shopping has been done by our children.’ Another patient wrote ‘Daily life has changed quite a bit due to corona. I stay indoors as much as possible’. Few patients also noted that they felt isolated, ‘Loneliness because of corona. I am unable to receive visitors and all other activities have been discontinued.’

## Discussion

This study showed that the COVID-19 pandemic did not affect the self-reported mental health of dialysis patients, as measured with HRQoL questionnaires. Dialysis patients did not report a higher burden or a higher severity of mental health-related symptoms such as feeling anxious, feeling sad, worrying, feeling nervous, trouble falling asleep, and trouble staying asleep.

A possible explanation for our findings could be that dialysis patients already suffer greatly from their kidney disease and treatment, which could limit the impact of the COVID-19 pandemic. Dialysis has a major impact on the mental health of dialysis patients resulting in a lower HRQoL than patients with other chronic illnesses such as malignancies [19, 20]. Mittal et al. [20] found that patients with kidney disease had a 2.68 point lower MCS score compared to the general population, whereas patients with malignancies had a 0.31 lower MCS score compared to the general population. In addition, dialysis patients have to deal with fluid restrictions, polypharmacy, and frequent hospital visits. As a result, dialysis patients have to adjust their everyday life for they encounter all these difficulties and adversities. As such, they have developed coping mechanisms in order to maintain satisfactory mental health. This ability to adapt is called resilience in literature and is often described as ‘a measure of successful stress-coping ability’ [21]. Resilience includes having a positive perception, accepting a burdensome situation, and being motivated to overcome various difficulties [22]. In a Spanish study, a higher level of resilience was associated with higher HRQoL scores [23]. The importance of resilience for both haemodialysis and peritoneal dialysis patients to overcome the burden of dialysis has been emphasized in multiple studies [24–26]. In one of these studies the resilience of dialysis patients was quantified with a frequently used resilience scale. They found a score of 82.4 in dialysis patients, comparable to the general population (80.4) and reasonably higher than among patients visiting a general practitioner (71.8) [21, 24]. Dialysis patients may have a high level of resilience compared to primary care patients, as they have learned to adapt over time to bear the burden of dialysis and their disease in general, which could explain their ability to deal better with different stressors such as the COVID-19 pandemic.

The large amount of unemployed dialysis patients in our population may also explain why the COVID-19 pandemic did not seem to affect mental health. A study showed that people who are unemployed had higher mental distress in general, but did not experience an increase of mental distress during the COVID-19 pandemic as assessed with the 12-item General Health Questionnaire (change score − 0.48 (95% CI − 1.55 to 0.60) [27]. Whereas people who are employed during the COVID-19 pandemic experienced an increase in mental distress compared to the period before COVID-19 (change score 0.63 (95% CI 0.20–1.06) [27, 28]. In our population only 16% was employed, which is consistent with clinical practice as many dialysis patients are unemployed.

The third possible explanation for our results could be that 75% of our study population received in-centre haemodialysis, which might diminish mental problems that could have developed as a result of the national social isolation. Support from fellow patients, nurses, and health care professionals can contribute to a reduced sense of loneliness. Moreover, dialysis patients usually participate less in everyday activities than age-matched healthy individuals or even kidney transplant patients due to the nature of the dialysis treatment [29]. The regular visits to the hospital for dialysis treatments consumes an important part of the patient’s time, with less time for social activities, work or travelling. Dialysis patients will be affected less by national policy measures such as social distancing since they experience fewer major changes in everyday life. In addition, in-centre haemodialysis patients might experience a sense of safety during their hospital visits that further limits the effect of the COVID-19 pandemic on mental health. In the Netherlands, many precautionary measures were taken at dialysis centres, such as screening for fever/complaints at entry for all patients, distance of 1.5 m whenever possible between people and wearing of face masks for dialysis patients, dialysis nurses and physicians early in the course of the pandemic. Also, dialysis patients that attended the hospital for haemodialysis sessions were able to obtain adequate information concerning COVID-19 directly from their health care professionals. In a Chinese study it was found that more information about the disease contributed to less anxiety levels [4].

It should be noted that some dialysis patients did express feelings of loneliness due to social isolation in the additional comments of the questionnaire. Because of their vulnerability they were being extra careful to protect themselves; informal caregivers took over many tasks for the patients so that they could avoid contact with others as much as possible. In a national survey among the general Dutch population, more than half of the participants indicated moderate or severe feelings of loneliness from April to June 2020. Nonetheless, they found that concerns among the general Dutch population began to subside around the end of March 2020 [30]. At this point the number of newly reported corona cases also began to decline. Compared to other countries in Europe including France, the United Kingdom and Italy, the number of newly reported COVID-19 patients and deaths was lower in the Netherlands, which could be a final explanation of the results in our study [31].

The results of our study are in line with a recent study in the United Kingdom, which showed that the COVID-19 pandemic did not affect the mental health of patients with chronic illnesses as assessed with a generic HRQoL questionnaire (change score in the GHQ-12 0.40 (95% CI − 0.30 to 1.09) [27]. Contradictory, an online survey among 1210 Chinese people found higher levels of stress, depression, and anxiety among those with a history of chronic illnesses [4]. Another study conducted in Northern Spain also showed higher levels of stress, depression, and anxiety among those with a history of chronic illnesses [5]. Unfortunately, none of all these studies specified the participants’ diseases, making a good comparison with our study population difficult. Also, in two studies no comparison with a historic control group or a pre-COVID-19 assessment of mental health was performed [4, 5].

To our knowledge, this is the first study investigating whether the COVID-19 pandemic affects the mental health of dialysis patients. Strengths of this study include the use of validated self-reported HRQoL questionnaires and the use of an existing prospective and nationwide cohort of dialysis patients (DOMESTICO) for analysis. Moreover, the number of patients in our study would have been sufficient to detect a significant difference in SF-12 composite scores between time periods as small as 2.17, whereas a difference of 3 is defined clinically relevant in literature [17, 18]. We calculated in our sample size that a total of 123 patients was sufficient to detect a 3 point difference between time points (*α* = 0.05, *β* = 0.10). In our study, we had a 97% power to detect such a clinically relevant difference. A limitation of our study might be that the MCS score of the SF-12 questionnaire is not sensitive enough to detect differences over time in individuals, i.e. that the MCS score has limited responsiveness [32]. To overcome this issue, we also used the DSI which provides more detailed information about the mental health of the patients. Another limitation might be that the chosen period of the COVID-19 pandemic was too short to demonstrate an association with mental health. The COVID-19 virus is still spreading and its effect on the economy is currently unclear. Therefore, if the pandemic lasts longer, a negative impact on mental health may still be revealed.

In conclusion, the mental health of dialysis patients assessed with SF-12 and DSI appears to have been unaffected during the first wave of the COVID-19 pandemic. This could be explained by higher resilience, more unemployment among dialysis patients, less impact of social distancing on the dialysis population, strict precautionary measures and perceived support from health care professionals, which may all contribute to better coping with the COVID-19 pandemic. However, a second peak of COVID-19 is expected and the economic burden of the pandemic has yet to be discovered. Therefore, it is important to continue paying attention to the concerns and needs of our dialysis population.

## Supplementary Information

Below is the link to the electronic supplementary material.

Supplementary file1 (PDF 264 KB)

## Data Availability

The data underlying this article are subject to an embargo of twelve months after completion of the DOMESTICO study. Once the embargo expires the data will be available upon reasonable request.
