# Supplementary material for: The Impact of COVID-19 on the mental health of dialysis patients
Source: J Nephrol. 2021 Mar 19;34(2):337–44. doi: 10.1007/s40620-021-01005-1 (PMC7978448; doi:10.1007/s40620-021-01005-1)
Supplement: Supplementary file 1 — Supplementary file1 (PDF 264 KB) [file 40620_2021_1005_MOESM1_ESM.pdf]

Supplementary figure for 'THE IMPACT OF COVID-19 ON THE MENTAL HEALTH OF DIALYSIS PATIENTS' in Journal of Nephrology

Anna A. Bonenkamp[1] MD, Theresia A. Druiventak[2], Anita van Eck van der Sluijs[2] MD, Frans J. van Ittersum[1] MD PhD, Brigit C. van Jaarsveld[1,3] MD PhD and Alferso C. Abrahams[2] MD PhD on behalf of the DOMESTICO study group

[1] Department of Nephrology, Amsterdam UMC, Vrije Universiteit Amsterdam, Research Institute Amsterdam Cardiovascular Sciences, Amsterdam, Netherlands [2] Department of Nephrology and Hypertension, University Medical Centre Utrecht, Utrecht, the Netherlands [3] Diapriva Dialysis Centre, Amsterdam, the Netherlands

Corresponding author
Name: Alferso C. Abrahams
E-mail address: A.C.Abrahams@umcutrecht.nl

Supplementary Figure 1. Severity of mental health-related symptoms prior and during COVID-19 pandemic

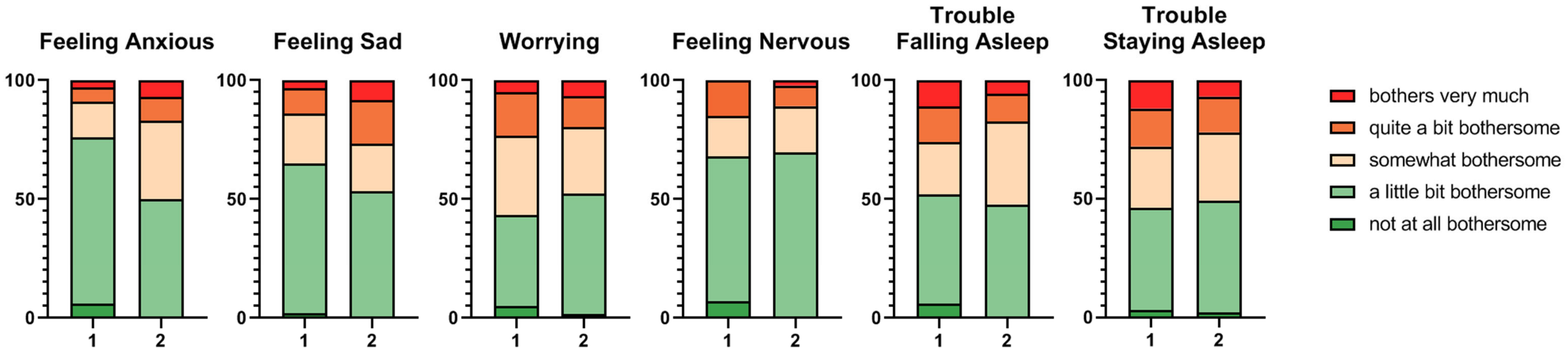

1; period prior to COVID-19 pandemic. 2; period during COVID-19 pandemic.
